# Video Game Playing Effects on Obesity in an Adolescent with Autism Spectrum Disorder: A Case Study

**DOI:** 10.1155/2015/128365

**Published:** 2015-12-10

**Authors:** Brandy E. Strahan, Jennifer H. Elder

**Affiliations:** ^1^University of West Florida, 11000 University Parkway, Building 37, Pensacola, FL 32514, USA; ^2^University of Florida, College of Nursing, P.O. Box 100187, Gainesville, FL 32610-0187, USA

## Abstract

Adolescent obesity has tripled in the past two decades, and adolescents with disabilities, specifically autism spectrum disorders (ASD), may be at greater risk for obesity due to the behavioral, physical, and psychosocial complications related to their disorder. This case study reports the effects of video game playing on an obese adolescent with ASD and illustrates the use of a multiple baseline single subject design. Over 12 weeks, the participant played inactive (6 weeks) and active video games (6 weeks) on the Wii console. Physiological data were evaluated weekly at home. Stress and anxiety were measured via the Stress Survey Schedule for Individuals with Autism and Other Pervasive Non-Developmental Disorders (SSS) and the Behavior Assessment System for Children Second Edition (BASC-2) pre- and postintervention. The Therapy Attitude Inventory (TAI) was used to determine parental perception of video game playing as a socially valid intervention to reduce stress and anxiety. Results demonstrated that active video game playing slowed and/or reduced weight and BMI with minimal changes to waist-to-hip ratios, triceps skinfolds, and stress and anxiety. This study demonstrates how alternative methods for physical activity may be used to improve health outcomes of overweight/obese adolescents with ASD and suggests directions for future research.

## 1. Introduction

Adolescent obesity has tripled over the past two decades across all race and ethnic groups, genders, and socioeconomic classes creating an epidemic and public health crisis [1]. Indeed adolescent obesity can contribute to serious health problems such as depression, diabetes mellitus, sleep apnea, and cardiovascular disease as well as social problems such as isolation, stigma, discrimination, and low self-esteem [2, 3]. Furthermore because nearly 70% of obese adolescents continue the trend into adulthood, [4, 5] consequences to their health are likely to be long term [6].

Children with developmental disabilities, such as those with autism spectrum disorders (ASD), may be particularly vulnerable to the development of obesity due to the behavioral, physical, and psychosocial complications related to their disorder [2, 7]. Indeed, children with ASD have a higher prevalence of obesity (30.4%) compared to children without ASD (23.6% [2]). Although numerous studies address obesity in typically developing adolescents, none address the problem among the ASD population.

ASD presents unique challenges for researchers and warrants novel interventions that can be tailored to meet the needs of individuals to address the challenge of obesity. Many caregivers of adolescents with ASD are often focused on problematic ASD features, with less concern for adverse health behaviors and weight [8], aberrant eating patterns and atypical physical activity [2], and/or excessive consumption of junk food [2, 9]. Although exercise has been shown to reduce stress and anxiety, which may be exhibited as tantrums in the ASD population, it may not be encouraged due to motor impairments, including poor motor skills, uneven developmental milestone acquisition, low muscle tone, and postural instability, frequently leading to decreased physical activity [2, 9]. Inactivity is not the only contributor to weight gain in children with ASD. Medications, such as Risperdal, often used to treat common features of ASD (such as self-injurious behavior, mood swings, and aggression towards others) have weight gain as a side effect [10]. Obesity is estimated to be 40% more likely in adolescents with ASD [2] than their typically developing peers and is rising at a rate that will outpace that of the general population [3].

Since nearly three-quarters of obese adolescents with ASD continue the obesity trend into adulthood, many develop comorbidities that are beyond the scope of previous interventions [5]. Additionally, young adults with ASD have a decrease in physical activity and become lethargic perpetuating the vicious cycle of caloric intake exceeding output. Indeed, one study of young adults with ASD found the mean sitting time in one day was 13 hours and that the lack of physical activity was due to disability; cost of equipment; lack of energy, leader, or partner, and athletic ability [5]. Clearly, there is a need to develop an intervention targeting the problem of obesity in adolescents with ASD.

One such creative approach is the use of electronic video games as an intervention to treat obesity in adolescents with ASD. Maddison et al. [11] conducted a randomized controlled trial with typically developing adolescents who were overweight or obese (*N* = 322) and were sedentary video gamers in order to evaluate the effects of physically active video games on participants' weight, body composition, physical activity, and fitness. Results revealed that the intervention group had a slight change in body mass index (BMI) from baseline (*p* = .02), a reduction in body fat (*p* = .02), and an increase in active video game play time (*p* < .01). In contrast, adolescents in the control group continued to gain weight playing inactive video games. These findings indicate that creative interventions have the potential to slow weight gain, an important component in reducing obesity.

Physical activity interventions represent an effective method to induce weight loss in adolescents with ASD. Active video gaming could have an effect on obesity in male adolescents with ASD since 41% of their free time is spent playing video games [12] and they may willingly adopt an active version of their favorite game or activity [11]. Thus, electronic video gaming provides an innovative alternative approach for treatment of obesity in adolescents with ASD that can be adapted to the individuals' preference of games and activities.

There are no studies to date that have examined the effects of video game playing on obese adolescents with ASD. Thus, the purpose of this case study was to determine the feasibility and effectiveness of active video game playing for an obese adolescent with ASD.

## 2. Method

The Institutional Review Board at University of Florida in Gainesville approved this work. The participant, “JD,” was a Caucasian male adolescent with mild-to-moderate ASD, diagnosed at the age of 5, and was 15 years of age. JD's BMI classified him as obese, 28.7 kg/m^2^, as defined by the CDC, and had other medical diagnoses such as Attention Deficit/Hyperactivity Disorder (ADHD) and Obsessive Compulsive Disorder (OCD). His daily medication regimen included Intuniv, 3 mg per day, Clonidine, 0.15 mg at night, Prozac, 20 mg per day, Trazadone, 75 mg at night, Clemastine, 0.67 mg in the morning and 4.02 mg at night, probiotics, and a multivitamin. He lived with his mother and a sibling and had weekend visitation with his father. JD was enrolled in public school and did not participate in any extracurricular activities. He typically stayed home and socialized with his family and a neighbor boy who is four years younger than JD. His diet consisted of two bowls of cereal or muffins for breakfast, pizza or sandwich with chips for lunch, and chicken nuggets, pasta, or pizza for dinner. Although his family offered healthier selections, he consistently chose these items.

### 2.1. Video Game Intervention

JD and his parents were told that they were participating in a video game playing intervention to learn more about exercise via active video game playing and weight in adolescents with ASD. JD and his mother were blinded to the study hypotheses and were told not to change any habitual physical activities or eating habits over the course of the study.

The active video game playing was completed using the Wii console and accessories needed. JD was given gaming options rated “E” for everyone for the Wii console. He chose an inactive and an active game. Baseline data were collected for four weeks prior to any gaming. JD's physiological data (height, weight, BMI, triceps skinfold, and waist-to-hip ratio) were collected during baseline and gaming weeks while gaming data (activity steps and playing time) were collected each week during interventional weeks of the study. JD also kept a daily food and activity log during all weeks of the study. After collection of baseline data, JD was instructed to play the chosen inactive video game for four or more days a week for a minimum of 30 minutes each day for a total of six weeks. Then, he played an active video game for four or more days a week for a minimum of 30 minutes each day for a total of six weeks.

### 2.2. Weight

JD was weighed at the same time of day using the same digital scale. Weight was obtained once a week during baseline and intervention phases in his home. JD wore similar clothing for weight assessment during the entire study.

### 2.3. Body Mass Index (BMI)

Body mass index (BMI) was calculated using JD's height and weight as defined by the CDC [13] and obtained weekly during baseline and intervention phases. Height was measured in inches with JD standing flat footed with no shoes and his weight was measured in pounds. Height was then converted to meters and weight to kilograms (weight/height^2^). Once the BMI was calculated, his weight status was determined using an age- and sex-specific percentile for children aged 2–19 years. Overweight was defined as “a BMI at or above the 85th percentile and lower than the 95th percentile for children of the same age and sex” and obese as “a BMI at or above the 95th percentile for children of the same age and sex” [13].

### 2.4. Waist-to-Hip Ratio

Waist-to-hip ratio was measured in inches using the same measuring tape and collected weekly during baseline and intervention phases. The ratio provides an indication of the overall health risk indicating low, moderate, or high risk for developing diseases such as cardiovascular disease or diabetes. Ratio norms are different for girls and boys, thus removing gender bias. Waist-to-hip ratio was obtained by measuring around the smallest part of the waist (just above the naval) and the widest part of the hips without pants or shorts but over underpants.

### 2.5. Triceps Skinfold

Anthropometric data, triceps skinfolds, were measured on the right arm using Harpenden calipers. Measurements were obtained weekly during baseline and intervention phases. Triceps skinfolds have been shown to more adequately correlate body fat percentages for children and adolescents while height and weight are better instruments for total body fat percentages [14].

### 2.6. Stress and Anxiety

Stress and anxiety were measured using the Stress Survey Schedule for Persons with Autism and Other Developmental Delays (SSS [15]) and Behavior Assessment System for Children-Second Edition (BASC-2 [16]) and completed by the same parent pre- and postintervention to assess stress and anxiety of JD. The SSS is a survey of 49 items rated on a Likert scale where 1 indicates none to mild and 5 indicates severe; the eight subscales range in reliability from 0.57 (the smallest subscale of social/environment interactions with three items) to 0.91. All subscales other than social/environment were above 0.75 for reliability, indicating that the SSS is a valid tool for assessing perceived stress in persons with autism [17]. Currently, the SSS is the only tool available to assess stress specifically in the target study population. Social validity was determined through Q-sort surveys among autism professionals, specifically the Autism Society of America's Panel of Professional Advisors, and results revealed that 94% of the stressors identified in the SSS are relevant to individuals with ASD [17]. The BASC-2 contains three forms (teacher, parent, and self) that can be used individually or in any combination. Due to the developmental issues associated with an autism diagnosis, this study included the parent form. It has 160 items and can be completed in 20–30 minutes. Results are reported in four categories, each with additional subcategories. The following are the categories and subcategories for the BASC-2 parent form: externalizing problems (hyperactivity, aggression, and conduct problems), internalizing problems (anxiety, depression, and somatization), behavioral symptoms index (atypicality, withdrawal, and attention problems), and adaptive skills (adaptability, activities of daily living, leadership, social skills, and functional communication). These results are reported as potential problem areas as clinically significant or at risk if the scores fall within a particular range. Reliability for the composite scores is 0.90, and test-retest reliability is 0.80. Validity was determined through parental, teacher, and psychologists' content as well as diagnostic criteria from the DSM-IV and DSM-IV-R [18] and has been used in previous studies of children with ASD [19, 20].

### 2.7. Therapy Attitude Inventory (TAI)

Parental satisfaction with the video game playing intervention was measured by the Therapy Attitude Inventory (TAI [21]). The TAI is a 10-item questionnaire of parental satisfaction with the adolescent's behavior changes and type of treatment used. JD's parent was asked to rate each item on a 5-point scale where 1 indicates dissatisfaction or worsening of problems and 5 indicates maximum satisfaction. The scores are summed to yield a total score between 10 and 50. Cronbach's alpha has been reported at 0.91 [21]. The TAI was obtained at the conclusion of the intervention.

### 2.8. Procedure

JD and his mother provided assent and written informed consent, respectively. A pretreatment session lasted approximately one hour in which basic information, such as demographic and medical information, was collected. Following the pretreatment assessment, JD completed four baseline assessments for a level of experimental control to demonstrate change as a result of treatment. Each baseline session lasted approximately 30 minutes and consisted of weekly measurements of weight, BMI, waist-to-hip ratio, and triceps skinfolds. JD's mother also completed stress and anxiety surveys during the first session.

**Figure 1:**
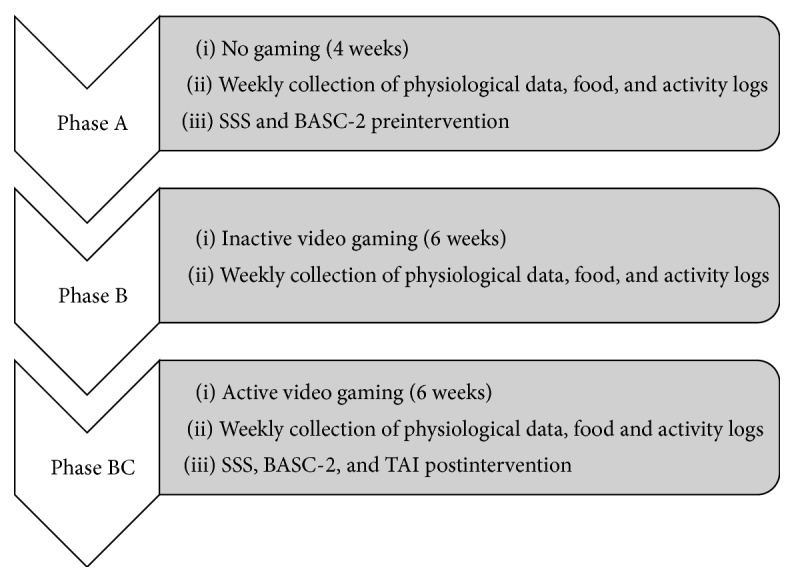
Procedure for baseline and interventional phases.

Following baseline sessions, JD was enrolled in treatment. The protocol involved 6 weeks of inactive video game playing for four days a week and a minimum of 30 minutes each day, followed by 6 weeks of active video game play for the same amount of time and days. Video games selected for the study were rated E (Everyone) by the Entertainment Software Rating Board and were considered suitable for ages of 6 years and older. Selection was based on current popularity, activity levels, and review of market ratings. During video game play, JD wore a Fitbit, a wireless tracker in a wristband that tracks steps taken, distance traveled, calories burned, and very active minutes, to ensure video game play as well as documenting video game play on their activity logs. The Fitbit was chosen for its convenience of wear, wrist only, and its proven reliability and validity of measuring step counts and energy expenditure in previous research [22–24]. Measurements of weight, BMI, waist-to-hip ratio, and triceps skinfold collected weekly during both inactive and active game playing phases (see Figure 1).

After completing all phases, JD's mother completed the stress and anxiety surveys as well as the TAI to determine social validity of the intervention and their perception of its effectiveness. Furthermore, feedback regarding barriers to performing the intervention and the feasibility of continuing a similar treatment to address obesity and health issues were discussed.

## 3. Results

**Figure 2:**
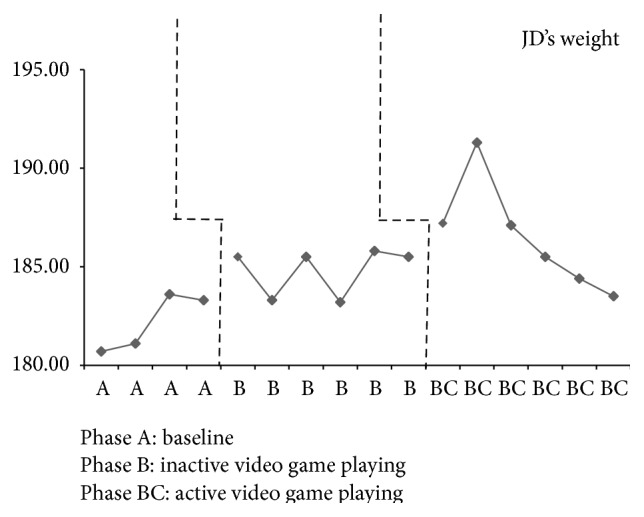
JD's weight across all phases.

**Figure 3:**
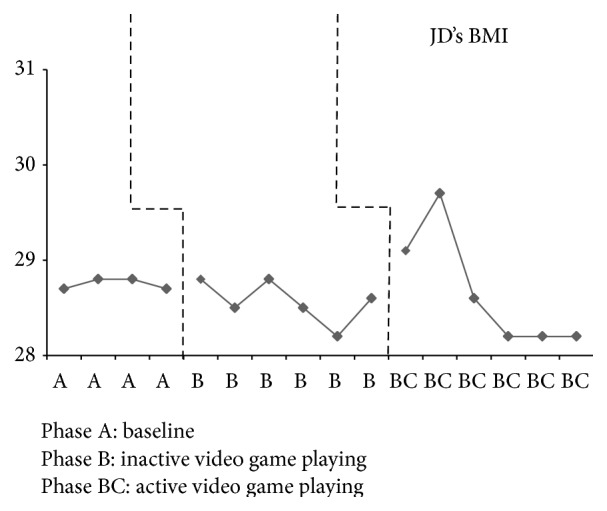
JD's BMI across all phases.

**Figure 4:**
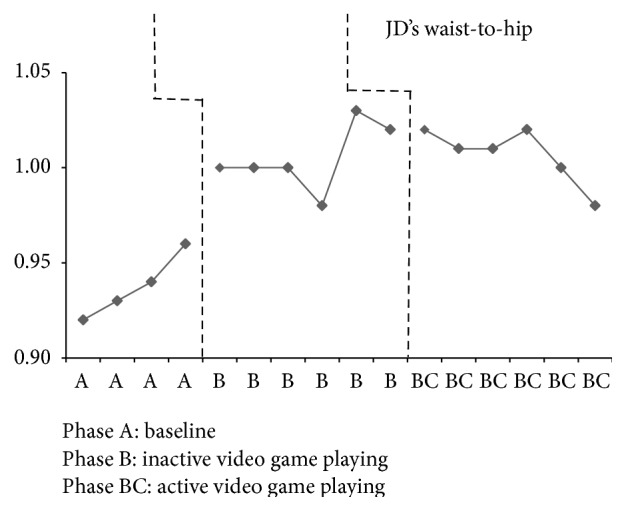
JD's waist-to-hip ratio across all phases.

**Figure 5:**
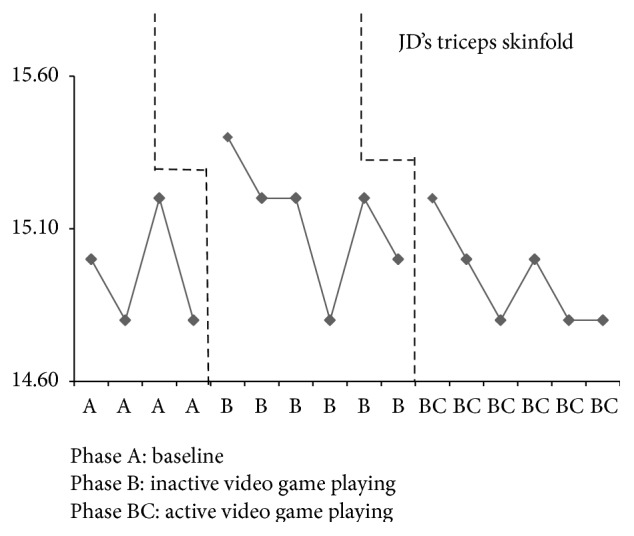
JD's triceps skinfolds across all phases.

JD had four baseline assessments that resulted in an increase in weight every week except the final baseline week, when there was a loss of 0.30 pounds (see Figure 2). Inactive video game playing, phase B, results demonstrate somewhat of a steady state in which his weight varied by 0.20 to 0.30 pounds in all but two weeks. JD's weight dropped by 2.20 pounds the second week and 2.30 pounds the fourth week of inactive video game playing. He increased in weight during the first two weeks of active video game playing by 1.70 pounds and 4.10 pounds; however, the remaining weeks of this phase resulted in weight loss by 0.90 pounds to 4.20 pounds. The percent of weight change for JD from the final baseline assessment (phase A) to the end of inactive video game playing (phase B) was a 1.2% increase and for active video game playing (phase BC) was a 0.1% increase. BMI for JD remained in a stable state during baseline with a variation of only 0.01 kg/m^2^ and in phase B a variation of only 0.30 to 0.40 kg/m^2^ (see Figure 3). Active video game playing, phase BC, resulted in an increased BMI for the first two weeks and then a decrease for all remaining weeks. JD demonstrated minimal changes across all phases for waist-to-hip ratios with a range of 0.01 to 0.04 (see Figure 4). Similarly, triceps skinfold measurements were fairly consistent across all phases with a range of 0.20 to 0.40 mm (see Figure 5).

Preintervention results of stress and anxiety for JD demonstrated moderate-to-severe anxiety with the SSS instrument in the following categories: changes, unpleasant experiences, sensory/personal, food related, and rituals. Results for moderate anxiety were in the categories of anticipation, positive experiences, and social/environment. The preintervention results for the BASC-2 were noted to have clinically significant *T* scores in the following categories: externalizing problems (hyperactivity), internalizing problems (composite score, anxiety, and depression), behavioral symptoms index (composite score, atypicality, withdrawal, and attention problems), and adaptive skills (composite score, activities of daily living, leadership, and functional communication). The BASC-2 preintervention *T* scores that were classified as at-risk were externalizing problems (composite score) and adaptive skills (adaptability). Postintervention results for the SSS only had minimal changes that did not result in a difference in categorical classification. Postintervention BASC-2 results for JD did not result in any changes in *T* scores. The TAI results for JD revealed a score of 42 out of 50, demonstrating that the parents perceive active video game playing as an effective intervention in reducing stress and anxiety.

## 4. Discussion

JD had reductions in weight after the introduction of active video game playing; however, there was an overlap of data between phases. For example, JD's waist-to-hip ratios between finishing phase B and starting phase BC were the same measurement. Interestingly, JD gained weight after the introduction of the intervention designed to increase physical activity. As reported on weekly food logs and verbally by his mother, JD increased his food intake during the first few weeks of increased physical activity, which may account for the increase in weight gain. Furthermore, foods chosen to offset the caloric expenditure were the typical energy dense favorites: chicken nuggets, pizza, and hot dogs. An additional explanation for the weight gain after the introduction of the active video game may be an increase in muscle mass. However, after these first few weeks, JD continued to reduce his weight as well as BMI, even if minimally. Although JD's overall weight increased, the active video game playing slowed the progression of weight gain during phase BC. BMI also decreased due to changes in weight and height during the intervention. Similar to Maddison et al. [11], these results demonstrate that physically active video games may be a valid option to produce weight loss which could reduce the likelihood of serious health problems such as diabetes, cardiovascular disease, and sleep apnea as well as the financial strain of obesity. Along with improved health, the significance of reducing and/or slowing weight gain among overweight/obese adolescents with ASD is that parents can keep the focus on the problematic features of ASD rather than the weight. The waist-to-hip ratio demonstrated that JD was at moderate-to-high risk for the typical serious health problems associated with obesity. Although waist-to-hip ratio and triceps skinfolds did not have significant changes, the active video game playing slightly decreased those measurements over a six-week period of time which may slow the insidious progression of health problems associated with obesity.

Stress and anxiety results had minimal changes in pre- and postintervention phases demonstrating that physically active video game playing did not reduce stress or anxiety in all areas. Although these findings were counter to expectations, research has shown that approximately 40% of children under the age of 18 with a diagnosis of ASD also had at least one comorbid anxiety disorder [25]. Thus, stress and anxiety coexist within this population beyond the typical stressors and may not significantly improve with physical activity. Interestingly, his mother reported improved behavior with siblings and parental interactions, indicating added benefits of active video game playing and explaining parental satisfaction TAI scores.

Although the results from the case study are preliminary, there is a need for future research to address limitations and replicate findings. A clear limitation of this work is that it describes only a single participant and that these findings may not be generalizable. However, single subject design serves a distinct purpose in that it allows for exploration of individual patient characteristics on treatment outcomes and an opportunity to manipulate variables during intervention. Additionally, the present study results may have been influenced by expectations; that is, JD and his family were aware of active video game playing even though they were blinded to the hypotheses. This may have contributed to positive findings as JD and his mother may have consumed improved food choices and/or less food to produce weight loss. However, results obtained by multiple baseline design suggest that positive outcomes are less likely to explain treatment effects since changes were directly related to the intervention. The length of the intervention may be a limitation because the physiologic data of waist-to-hip ratio and triceps skinfold measurements may not demonstrate changes over short periods of time. Another possible limitation is that the stress and anxiety measures were based on parent report which has the potential for parental bias and may lack sufficient objectivity. Finally, JD demonstrated overlap of data between phases B and BC which may create difficulty in establishing a true functional relationship of variables. Therefore, study replication with extended baseline assessments and intervention phases is needed to determine if there is true cause and effect. Further research, including study replication and randomized controlled trials with larger samples of adolescents of both genders and varied ethnicities, is needed to determine if the treatment effects of active video game playing are robust and generalizable.

## 5. Conclusion

The case study report describes a protocol that can be replicated and provides preliminary evidence for the effectiveness of active video game playing with an overweight/obese adolescent with ASD. As noted previously, there are no studies yet published regarding the use of active video game playing in this population, a major gap in extant literature. Given the accumulating evidence for the effectiveness of physical activity in treating typically developing obese adolescents, it is critical that future research explores alternative solutions for physical activity among obese adolescents with ASD, for example, the active games used in this study.
